# Intestinally-restricted Janus Kinase inhibition: a potential approach to maximize the therapeutic index in inflammatory bowel disease therapy

**DOI:** 10.1186/s12950-017-0175-2

**Published:** 2017-12-06

**Authors:** David T. Beattie, M. Teresa Pulido-Rios, Fei Shen, Melissa Ho, Eva Situ, Pam R. Tsuruda, Patrick Brassil, Melanie Kleinschek, Sharath Hegde

**Affiliations:** 1 Department of Pharmacology , Theravance Biopharma US, Inc, 901 Gateway Boulevard, South San Francisco, CA 94080 USA; 2 Department of Biology, Theravance Biopharma US, Inc, 901 Gateway Boulevard, South San Francisco, CA 94080 USA; 3 Department of Drug Metabolism and Pharmacokinetics, Theravance Biopharma US, Inc, 901 Gateway Boulevard, South San Francisco, CA 94080 USA

**Keywords:** Inflammatory bowel disease, Tofacitinib, Janus kinase inhibitor, Pharmacokinetics, Pharmacodynamics

## Abstract

**Background:**

An unmet need remains for safe and effective treatments to induce and maintain remission in inflammatory bowel disease (IBD) patients. The Janus kinase (JAK) inhibitor, tofacitinib, has demonstrated robust efficacy in ulcerative colitis patients although, like other systemic immunosuppressants, there may be safety concerns associated with its use. This preclinical study evaluated whether modulating intestinal inflammation via local JAK inhibition can provide efficacy without systemic immunosuppression.

**Methods:**

The influence of tofacitinib, dosed orally or intracecally, on oxazolone-induced colitis, oxazolone or interferon-γ (IFNγ)-induced elevation of colonic phosphorylated signal transducer and activator of transcription1 (pSTAT1) levels, and basal splenic natural killer (NK) cell counts was investigated in mice.

**Results:**

Tofacitinib, dosed orally or intracecally, inhibited, with similar efficacy, oxazolone-induced colitis, represented by improvements in the disease activity index and its sub-scores (body weight, stool consistency and blood content). Intracecal dosing of tofacitinib resulted in a higher colon:plasma drug exposure ratio compared to oral dosing. At equieffective oral and intracecal doses, colonic levels of tofacitinib were similar, while the plasma levels for the latter were markedly lower, consistent with a lack of effect on splenic NK cell counts. Tofacitinib, dosed orally, intracecally, or applied to the colonic lumen in vitro, produced dose-dependent, and maximal inhibition of oxazolone or IFNγ-induced STAT1 phosphorylation in the colon.

**Conclusions:**

Localized colonic JAK inhibition, by intracecal delivery of tofacitinib, provides colonic target engagement and efficacy in a mouse colitis model at doses which do not impact splenic NK cell counts. Intestinal targeting of JAK may permit separation of local anti-inflammatory activity from systemic immunosuppression, and thus provide a larger therapeutic index compared to systemic JAK inhibitors.

## Background

Inflammatory bowel diseases (IBDs), such as ulcerative colitis and Crohn’s disease, have a significant, detrimental impact on the quality of life of patients [1]. Common IBD symptoms experienced during an active phase of the disease include diarrhea, rectal bleeding, abdominal pain, weight loss, fatigue, nausea and vomiting [2]. While a variety of therapeutic options exists to induce and maintain disease remission in IBD patients, each has its limitations. Aminosalicylates, often effective in mild disease, are much less so in moderate and severe disease, while long-term use of steroids for maintenance therapy has safety concerns (e.g., osteoporosis, muscle wasting, and neuropsychiatric disorders) [3]. Systemic immunosuppressants, such as azathioprine, mercaptopurine and methotrexate, often have modest efficacy in moderate/severe patients, and their prolonged use is problematic due to the consequences of long-term systemic immunosuppression (e.g., increased risk of infection and lymphoma) [4–6]. Anti-tumor necrosis factor (TNF) antibodies (e.g., infliximab and adalimumab) require subcutaneous or intravenous administration, and up to one third of patients fail to respond adequately, while another third of initial responders lose responsiveness due to the generation of antibodies to the drugs [7].

It is clear that an unmet medical need remains for an effective oral therapy to induce and maintain remission of IBD without the safety concerns resulting from chronic, systemic immunosuppression. Selective targeting of anti-inflammatory drug activity directly to the intestinal mucosa is an appealing means to achieve this objective, and several approaches appear feasible [8, 9]. Compounds with appropriate physicochemical properties, and optimized formulations have been developed, and mechanisms selective for the intestine have also been targeted. An inactive prodrug that undergoes enzymatic cleavage during gastrointestinal transit to generate an active parent drug is another option to target the intestine selectively. Examples of IBD therapies selectively targeting the intestine already exist. Upon rectal dosing to ulcerative colitis patients, an enema of mesalamine, the active moiety of sulfasalazine, flows retrogradely as far as the splenic flexure, and is efficacious against segmental colitis of the descending colon [10]. Systemic absorption is limited upon intrarectal administration of mesalamine to humans, and the side effects evident with orally dosed sulfasalazine are lacking [11, 12]. Another example of gastrointestinal targeting is provided by vedolizumab (Entyvio^®^), a selective α_4_β_7_-integrin antagonist, approved for the treatment of Crohn’s disease and ulcerative colitis. Its gut-targeted anti-inflammatory activity results from exclusive sequestering of gut-homing lymphocytes in the intestinal mucosa [13]. The safety profile of vedolizumab has been evaluated in over 3300 adults, and the incidence of infections and malignancies is similar to that noted with placebo [14].

As many of the pro-inflammatory cytokines elevated in ulcerative colitis (e.g., IL-6, IL-13, IL-15, IL-23 and IFNγ) and Crohn’s disease (e.g., IL-13, IL15, IL-22, IL-24 and IL-27) rely on the Janus kinase (JAK) family of tyrosine kinases for signal transduction [15, 16], it has been proposed that JAK inhibition may be beneficial in the treatment of ulcerative colitis and Crohn’s disease. Tofacitinib (Xeljanz^®^), an oral, systemically available, pan-JAK inhibitor approved for rheumatoid arthritis, had robust efficacy in Phase 2 and Phase 3 ulcerative colitis clinical trials [17, 18]. However, based largely on clinical data for the compound in rheumatoid arthritis patients, dose-limiting, systemically-mediated, adverse events may prove to be an issue, either acutely or chronically (e.g., increased cholesterol levels, opportunistic infections, neutropenia, lymphocytopenia, lymphoma and solid tumors) [19, 20]. While the potential benefit of JAK inhibition in IBD therapy is clear, it is uncertain whether clinical efficacy is driven systemically, or can be provided via localized, intestinal targeting. If selective intestinal JAK inhibition is sufficient for clinical efficacy, it may be possible to dissociate the beneficial anti-inflammatory activity of a JAK inhibitor from the potentially harmful, systemically-mediated activity. This topic is addressed in the current preclinical study. The efficacy of tofacitinib, dosed orally or intracecally, in a mouse oxazolone-induced colitis model was determined. This model shares many of the features of ulcerative colitis in patients; there is superficial inflammation of the colonic wall, consisting of edema and ulceration of the epithelial cell layer, and corresponding reductions in body weight and stool consistency, together with the presence of blood in the feces [21, 22]. Additionally, the ability of tofacitinib to inhibit oxazolone- or interferonγ (IFNγ)-induced phosphorylated signal transducer and activator of transcription1 (pSTAT1) elevation in the mouse colon was evaluated following oral or direct colonic administration. To assess systemic JAK target engagement, the effect of tofacitinib on splenic natural killer (NK) cell counts was studied. A reduction in circulating NK cells has been demonstrated in rats, monkeys and humans following tofacitinib administration, and provides a sensitive measure of systemic JAK target engagement [23, 24]. Consistent with the role of NK cells in innate and adaptive immune responses [25], tofacitinib-induced suppression of NK cell activation, maturation and function could impair the body’s ability to fight infection or respond to tumor formation [26]. However, as NK cell numbers appear to recover upon chronic dosing to humans, the clinical significance of the acute reduction in NK cell levels is unclear [27]. Colon, plasma and spleen levels of tofacitinib were measured in this study to allow pharmacokinetic/pharmacodynamic analysis, and to provide insight on its site of action.

## Methods

All in vivo experiments were conducted with adult, male or female Balb/C mice (15–29 g) at Theravance Biopharma US, Inc. in accordance with its Institutional Animal Care and Use Committee (IACUC) approved protocols. Tofacitinib was synthesized by Theravance Biopharma, US, Inc. and YouChemicals, Inc. (Shanghai).

### Oxazolone-induced colitis

Mice were anesthetized (2% isoflurane inhalation), and oxazolone (Sigma Aldrich; 150 μL, 4%, 4:1 acetone/olive oil) was applied to an area of shaved skin between the shoulder blades. Seven days later, animals were fasted overnight and then anesthetized (2–4% isoflurane inhalation). A 3.5-F catheter, filled with oxazolone (1%, 1:1 ethanol/water) or vehicle, was inserted rectally ∼4 cm, and 50 μL was injected. Three days of oral dosing of tofacitinib (twice (BID) or three times daily (TID)), or its vehicle (0.5% carboxymethylcellulose) were initiated one day prior to the intrarectal oxazolone challenge. Two days after the intrarectal oxazolone challenge, the disease activity index (DAI) was calculated by blinded experimenters, as the averaged total of three subscores: stool consistency (0 = normal, 2 = loose, 4 = diarrhea), gross bleeding (0 = absence, 2 = blood tinged, 4 = presence; Hemoccult II^®^ SENSA^®^), and weight loss (0 = none, 1 = 1–5%, 2 = >5–10%, 3 = >10–20%, 4 = >20%). For colonic dosing, a saline-filled sterile cannula (1 mm outer diameter) was implanted into the cecum of anesthetized (2–4% isoflurane inhalation), fasted mice. The cannula was secured by sutures, and exteriorized on the animal’s back. Five weeks later, colitis was induced by oxazolone.

A Student’s t-test compared the DAI score (and subscores) of the vehicle/vehicle and vehicle/oxazolone groups (*p* ≤ 0.05 defining statistical significance). A one-way ANOVA, with a Fisher’s LSD post hoc test, compared the scores of the vehicle/oxazolone and tofacitinib/oxazolone groups (p ≤ 0.05 defining statistical significance).

### Assessment of Oxazolone and IFNγ-induced pSTAT1 in mouse colon

Tofacitinib or vehicle (0.5% carboxymethylcellulose) was administered orally 3 h after intrarectal oxazolone (50 μL, 1%, 1:1 ethanol/water) dosing. One hour later, mice were euthanized by CO_2_ inhalation. Frozen colon samples were homogenized (5500 rpm for 60 s at 4 °C; Precellys^®^) in lysis buffer (50 mM Tris pH 7.5, 150 mM NaCl, 5 mM EDTA, supplemented with 1× complete Ultra mini protease inhibitors (Roche) and PhosSTOP (Roche)). Radioimmunoprecipitation assay (RIPA) buffer (Cell Signaling Technologies) was added 1:10 to the homogenate and samples were vortexed briefly prior to incubation on ice for 25 min. Homogenates were sonicated, and centrifuged (10,000 x g, for 10 min at 4 °C). Supernatants were stored at −80 °C until analyzed. The protein concentration of the tissue lysates was measured (Bradford assay), and samples were diluted to ~1 mg/mL total lysate protein in lysis buffer supplemented with RIPA buffer (1:10).

For IFNγ-induced pSTAT1 studies, tofacitinib or its vehicle (0.5% carboxymethylcellulose) was dosed orally (10 mL/kg), followed 5 min later by intrarectal dosing (0.2 mL) of 30% ethanol to breach the colonic epithelium. One hour later, IFNγ (2 μg, 0.1 mL/min) was infused intrarectally, and animals were euthanized a further one hour later. The colon was removed, rinsed, and snap-frozen on dry ice.

In some experiments, mice were euthanized by CO_2_ inhalation, and the colon was placed in oxygenated Krebs-Henseleit buffer (Sigma-Aldrich). Colonic segments were attached to two cannulae (Nalgene^®^; inner and outer diameters of 2 and 3 mm, respectively), for the in-flow and out-flow of oxygenated Krebs-Henseleit buffer (37°C). During treatment times, out-flow was prevented using a microclamp. Following equilibration of tissues for one hour, 0.1 mL of 30% ethanol was delivered intraluminally. Fifteen minutes later, the ethanol was replaced with Krebs-Henseleit buffer (0.1 mL), containing tofacitinib or vehicle. The Krebs-Henseleit solution was removed 45 min later, and replaced with 50 μL of IFNγ (20 μg/mL), with tofacitinib or vehicle, for a further 75 min, at which time the tissues were removed and snap frozen.

Levels of pSTAT1 were detected by ELISA. Plates (96-well) were coated with 0.3 μg STAT1 capture antibody (#9172; Cell Signaling Technology) in Dulbecco’s phosphate buffered saline (DPBS), at room temperature, overnight. The plates were then washed with wash buffer (DPBS with 0.05% Tween-20). Plates were blocked with 2% BSA in DPBS, washed, and 60 μL of the diluted tissue lysate added to each well. They were then incubated at 4 °C overnight, followed by washing, and incubation with 3 ng of pSTAT1 detection antibody (#612232; BD) in reagent dilution buffer (2% BSA in DPBS with 0.05% Tween-20) for 2 h at room temperature. The plates were washed and incubated for 1 h with a horseradish peroxidase-conjugated secondary antibody (anti-mouse IgG, #7076S; Cell Signaling Technology) in reagent dilution buffer. Subsequently, the plates were washed, and tetramethylbenzidine (TMB) substrate (Cell Signaling Technology) was then added to each well. STOP solution (Cell Signaling Technology) was added after 15–30 min of color development, and absorbance (450 nm, reference at 540 nm) was read.

Raw data (absorbance) were subject to baseline subtraction using the buffer controls on the ELISA plate, and individual data points for the compound-treated groups were normalized using mean values from the control groups (vehicle- and oxazolone (or IFNγ)-challenged) to calculate percent inhibition. The potency of tofacitinib was expressed as an IC_50_ value (GraphPad Prism™).

For immunohistochemical analysis of IFNγ-induced pSTAT1 expression, the colon was prepared in a “Swiss roll” configuration [28], and fixed in formalin. Immunohistochemistry was performed on two 4 μm tissue slices per colon.

### Measurement of Splenic NK cell numbers

Mice were dosed orally or intracecally with tofacitinib (15 mg/kg or 1 mg/kg BID, respectively), or vehicle (0.5% carboxymethylcellulose or saline, respectively) for 3 days. Spleens were harvested 30 min after the last dose of tofacitinib and crushed immediately for NK cell staining. Splenocyte samples were incubated with a fluorophore-labelled antibody (APC-CD49+; BD Biosciences), and the absolute number of NK cells determined by flow cytometry (Becton Dickinson LSR II). Lymphocytes were first distinguished from cell debris based on forward and side scatter properties (FSC-A and SSC-A). CD49+ NK cells were then gated on the CD49+/CD3- quadrant. A one-way ANOVA, with a Fisher’s LSD post hoc test, compared the NK cell numbers between groups (*p* ≤ 0.05 defining statistical significance).

### Bioanalysis of Tofacitinib concentrations

Plasma, colon and spleen samples were collected to determine the tofacitinib C_max_ (appropriate timepoints determined in prior experiments). Terminal blood samples were collected by cardiac puncture into K_2_EDTA, and plasma prepared by centrifugation (3270 x g at 5 °C). Frozen colon and spleen samples were added to bead homogenizer tubes, diluted 1:10 with acidified water, and homogenized (Precellys^®^; 6500 rpm (3 × 45 s), 4 °C). Tissue homogenates were stored at −80°C until determination of tofacitinib concentrations by LC/MS/MS. After thawing and vortexing, a 10 μL aliquot of plasma or tissue homogenate was diluted 5-fold in mouse plasma, and extracted with 200 μL of acetonitrile. The extract was centrifuged (2089 x g), and the supernatant was transferred and diluted (4-fold) in 5 mM ammonium acetate in water. For liquid chromatography, 15 μL was injected on to a Waters Atlantis T3 (C18, 3 μm, 50 × 2.1 mm) column, at a flow rate of 0.35 mL/min. Mobile phase A consisted of 5 mM ammonium acetate in water and mobile phase B of 5 mM ammonium acetate in acetonitrile. The mobile phase gradient started with 10% to 95% B from 0.25 to 1.5 min, held at 95% from 1.5 to 1.75 min, followed by a gradient of 95% to 5% B from 1.75 to 1.76 min and stopped at 2.3 min. The assay range for tofacitinib was from 1.16 to 5000 ng/mL.

## Results

### Oxazolone-induced colitis model

**Fig. 1:**
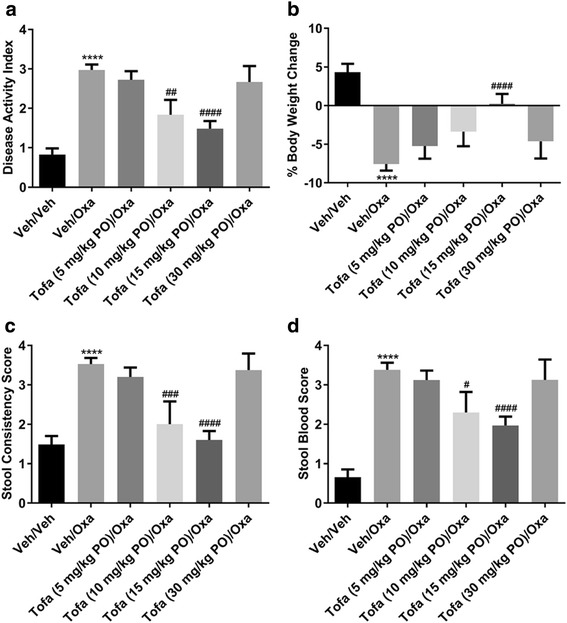
The effects of orally (PO) dosed tofacitinib (Tofa; 5–30 mg/kg TID) on the disease activity index (**a**), and its components, body weight (**b**), stool consistency (**c**) and occult blood (**d**), following oxazolone (Oxa) challenge to sensitized mice. Data are expressed as mean ± SEM (*n* = 8–35). **** *p* < 0.0001 vs. Veh/Veh (Student’s t-test), # *p* = 0.02, ## *p* = 0.003, ### *p* = 0.0008 and #### p < 0.0001 vs. Veh/Oxa (One-way ANOVA, Fisher’s LSD post-hoc test)

**Fig. 2:**
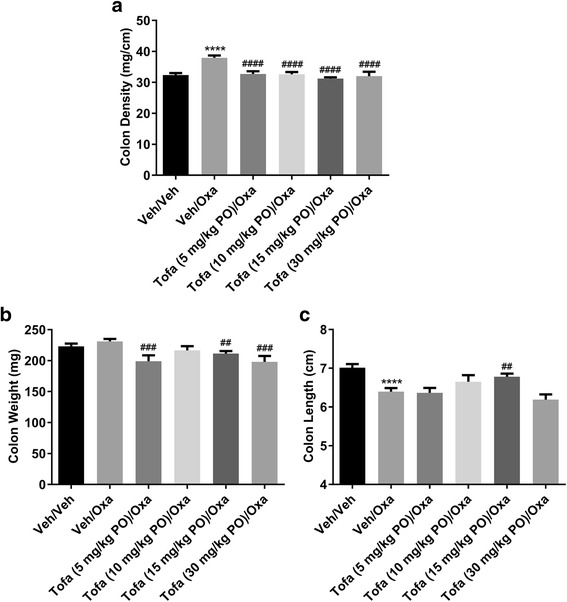
The effects of orally (PO) dosed tofacitinib (Tofa; 5–30 mg/kg TID) on (**a**) colon density, (**b**) weight (**b**), and (**c**) length following oxazolone (Oxa) challenge to sensitized mice. Data are expressed as mean ± SEM (*n* = 9–35). **** p < 0.0001 vs. Veh/Veh (Student’s t-test), ## p = 0.003 or 0.006, ### *p* = 0.0004, and #### p < 0.0001 vs. Veh/Oxa (One-way ANOVA, Fisher’s LSD post hoc test)

Intrarectal dosing of oxazolone (50 μL of 1% oxazolone) to sensitized mice resulted in an elevated DAI two days later compared to vehicle. With respect to the individual components of the DAI, oxazolone produced a significant reduction in body weight and stool consistency, and an increase in stool occult blood (Fig. 1). Oral dosing of tofacitinib (10 and 15 mg/kg TID) attenuated the oxazolone-induced, elevated DAI, reduction in body weight and stool consistency, and increase in stool blood content (Fig. 1). A bell-shaped dose response curve was evident; tofacitinib at the highest dose tested (30 mg/kg TID) had no statistically significant effect on DAI, stool consistency or stool blood (Fig. 1). Tofacitinib attenuated oxazolone-induced increases in colon density and decreases in colon length (Fig. 2).

**Fig. 3:**
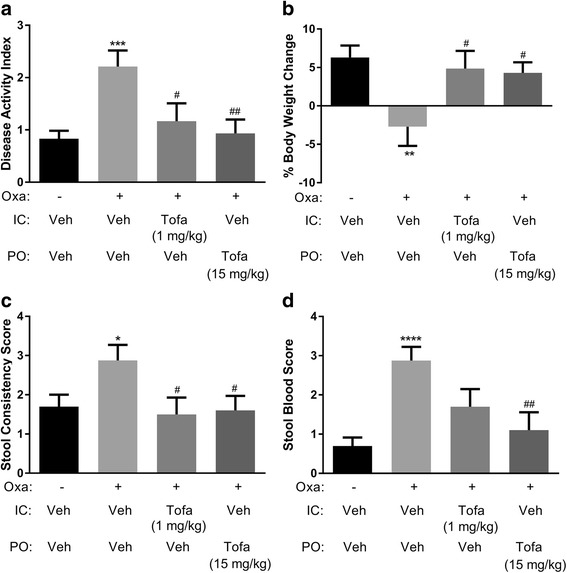
The effect of tofacitinib (Tofa), dosed intracecally (IC; 1 mg/kg BID, with accompanying oral (PO) vehicle dosing) or PO (15 mg/kg BID, with accompanying IC vehicle dosing) on the disease activity index (**a**), and its components, body weight (**b**), stool consistency (**c**) and occult blood (**d**), following oxazolone (Oxa) challenge to sensitized mice. Each control mouse received both PO and IC vehicles. Data are expressed as the mean ± SEM (*n* = 10–35). * *p* = 0.03, ** *p* = 0.006, *** *p* = 0.0006, and **** p < 0.0001 vs. Veh/Veh (Student’s t-test), # p = 0.02–0.04, and ## *p* = 0.009 vs. Veh/Oxa (One-way ANOVA, Fisher’s LSD post-hoc test)

**Fig. 4:**
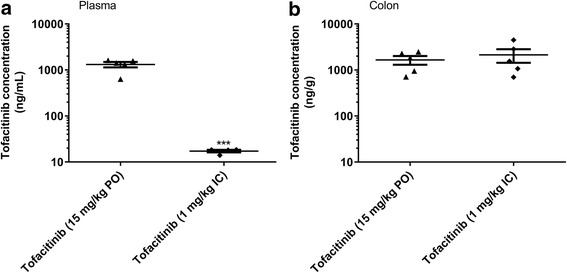
Plasma (**a**) and colonic (**b**) exposures of tofacitinib at its C_max_ (i.e., at 30 min post-dosing) following intracecal (IC; 1 mg/kg BID) and oral (PO; 15 mg/kg BID) administration of tofacitinib to oxazolone-treated mice (*n* = 4 or 5). Individual data points and the mean value (±SEM) for each treatment group are shown. *** p = 0.0004 vs. Tofacitinib (15 mg/kg PO; Student’s t-test)

Intracecally dosed tofacitinib (1 mg/kg BID) achieved a similar level of efficacy in the oxazolone colitis model to that following oral dosing (15 mg/kg BID; Fig. 3), and similar colonic exposures (mean concentrations (±SEM) at its C_max_ of 2128 ± 691 and 1656 ± 354 ng/g, respectively; Fig. 4). The 1 mg/kg intracecal tofacitinib dose, however, was associated with a significantly lower systemic exposure compared to that following the 15 mg/kg oral dose (mean C_max_ (±SEM) of 17 ± 1 and 1314 ± 179 ng/mL, respectively; Fig. 4). The calculated colon:plasma exposure ratios for tofacitinib were 1.3 and 125 following oral (15 mg/kg) and intracecal (1 mg/kg) dosing, respectively. Pharmacokinetic data from a separate study demonstrated that the plasma level of tofacitinib following oral dosing of 5 mg/kg TID was higher than that after intracecal administration (1 mg/kg BID); mean C_max_ values (±SEM) were 216 ± 37 and 17 ± 1 ng/mL, respectively, while the colon level was lower (306 ± 60 and 2128 ± 691 ng/g, respectively). At this lower oral dose, tofacitinib had no inhibitory effect on the oxazolone-induced effects on the DAI (Fig. 1).

### Oxazolone and IFNγ-induced pSTAT1 in mouse colon

**Fig. 5:**
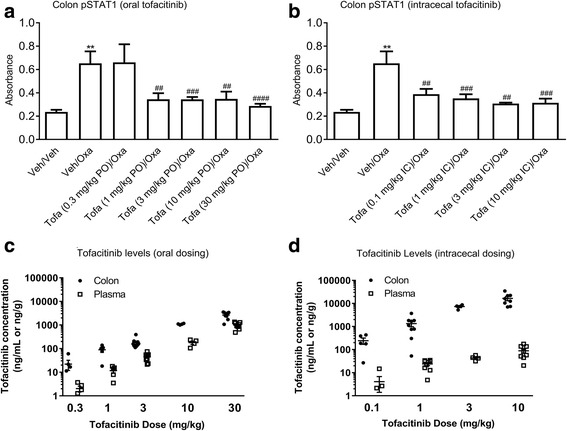
Effect of tofacitinib (Tofa), dosed (**a**) orally (PO; 0.3–30 mg/kg, with accompanying intracecal (IC) dosing of vehicle) or (**b**) IC (0.1–10 mg/kg, with accompanying PO dosing of vehicle), on oxazolone (Oxa)-induced pSTAT1 production in the mouse colon (mean ± SEM, *n* = 3–10). Each control mouse received both PO and IC vehicles. Corresponding colon and plasma concentrations of tofacitinib following (**c**) oral and (**d**) intracecal dosing are shown (individual data points and the mean value (±SEM); n = 4–9). ** *p* = 0.001 vs. Veh/Veh (Student’s t-test), ## *p* = 0.005–0.03, ### *p* = 0.0002–0.0005, and #### p < 0.0001 vs. Veh/Oxa (One-way ANOVA, Fisher’s LSD post-hoc test)

Intrarectal oxazolone administration increased colonic pSTAT1 levels 3-fold, 4 h after dosing (Fig. 5). The oxazolone-induced pSTAT1 response was inhibited by orally dosed tofacitinib (1–30 mg/kg) by up to 88 ± 5% (mean ± SEM) when dosed 3 h after intrarectal oxazolone (Fig. 5a). Similarly, tofacitinib dosed intracecally (0.1–10 mg/kg) inhibited the oxazolone-induced pSTAT1 elevation by up to 83 ± 2% (mean ± SEM) when administered 3 h after intrarectal oxazolone (Fig. 5b). Earlier dosing of tofacitinib, relative to oxazolone, had no effect suggesting the importance of dose timing in this acute target engagement model (data not shown). Intracecal dosing of tofacitinib resulted in higher colonic concentrations in comparison to those following oral administration, and a larger separation between colon and plasma levels (Fig. 5c and d). Pharmacokinetic/pharmacodynamic analysis indicated that doses of tofacitinib associated with significant inhibition of pSTAT1 elevation had mean (±SEM) colon levels of ≥89 ± 20 ng/g. The colon:plasma exposure ratios for intracecally dosed tofacitinib were markedly higher (i.e., 59, 56, 168 and 186 following the 0.1, 1, 3 and 10 mg/kg doses, respectively) than those following oral administration (i.e., 11, 8, 4, 6 and 3 for the 0.3, 1, 3, 10 and 30 mg/kg doses, respectively).

**Fig. 6:**
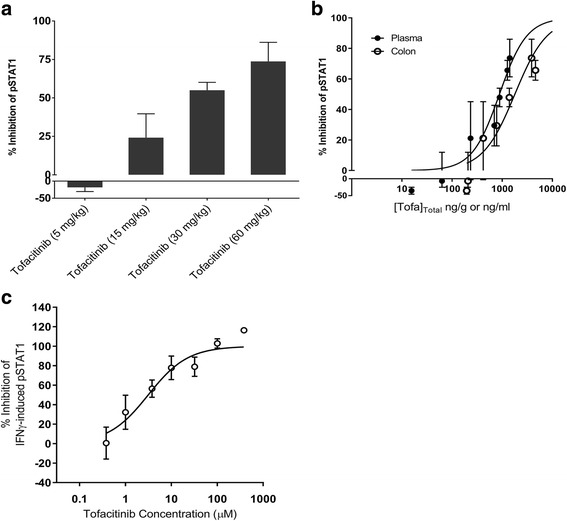
Effect of tofacitinib on intrarectally infused IFNγ-induced pSTAT1 production in mouse colon. Data (mean ± SEM, n = 4–11) are expressed with respect to (**a**) dose and (**b**) concentration. (**c**) Concentration/response curve to tofacitinib in the mouse isolated colon with respect to IFNγ-induced pSTAT1 production (mean ± SEM, n = 3–18)

In addition to the elevation of pSTAT1 by oxazolone, the ability of IFNγ to stimulated pSTAT1 was investigated in naïve mice. Intrarectal dosing of ethanol followed by IFNγ, but not its vehicle, resulted in an approximate 3-fold increase in colonic pSTAT1 levels, measured by ELISA, one hour after dosing. Tofacitinib (15–60 mg/kg orally) inhibited the IFNγ-induced pSTAT1 elevation in the colon in a dose-dependent manner (Fig. 6a). At the maximum oral dose tested (i.e., 60 mg/kg), tofacitinib inhibited pSTAT1 elevation by 74 ± 12% (mean ± SEM). Pharmacokinetic/pharmacodynamic analysis yielded concentration response curves with mean IC_50_ values (and 95% confidence intervals) of 1790 (975–3285) ng/g (colon) and 882 (561–1389) ng/mL (plasma) (Fig. 6b).

Following exposure to ethanol, IFNγ application (50 μL of 20 μg/mL) to the lumen of the mouse isolated colon produced an increase in pSTAT1 levels. Tofacitinib inhibited the IFNγ-induced pSTAT1 levels in a concentration dependent manner (Fig. 6c). The mean IC_50_ value (with 95% confidence intervals) was 3.1 (1.8–5.4) μM (equivalent to 968 (562–1687) ng/g).

**Fig. 7:**
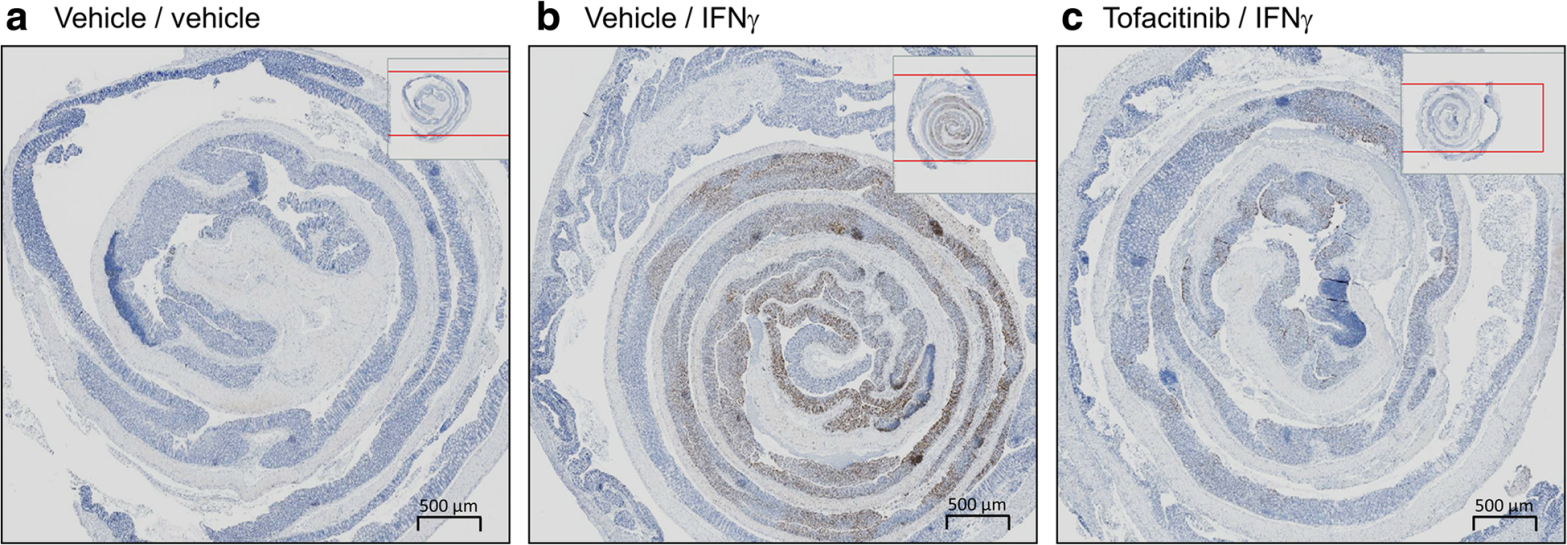
Representative images of colons from mice treated intrarectally with ethanol, and then challenged with vehicle or IFNγ, and stained immunohistochemically with a monoclonal antibody to pSTAT1. Mice were pretreated orally with vehicle followed by intrarectal vehicle (**a**), or IFNγ (**b**), or with tofacitinib (60 mg/kg orally) followed by intrarectal IFNγ (**c**). pSTAT1 is visually represented by the dark staining. The colon is coiled in a “Swiss roll” configuration

Immunohistochemistry analysis demonstrated that intrarectal dosing of IFNγ, but not vehicle, increased pSTAT1 expression across all layers of the colon (Fig. 7b). The pSTAT1 levels were inhibited by oral dosing of tofacitinib (Fig. 7c).

### Splenic NK cell numbers

**Fig. 8:**
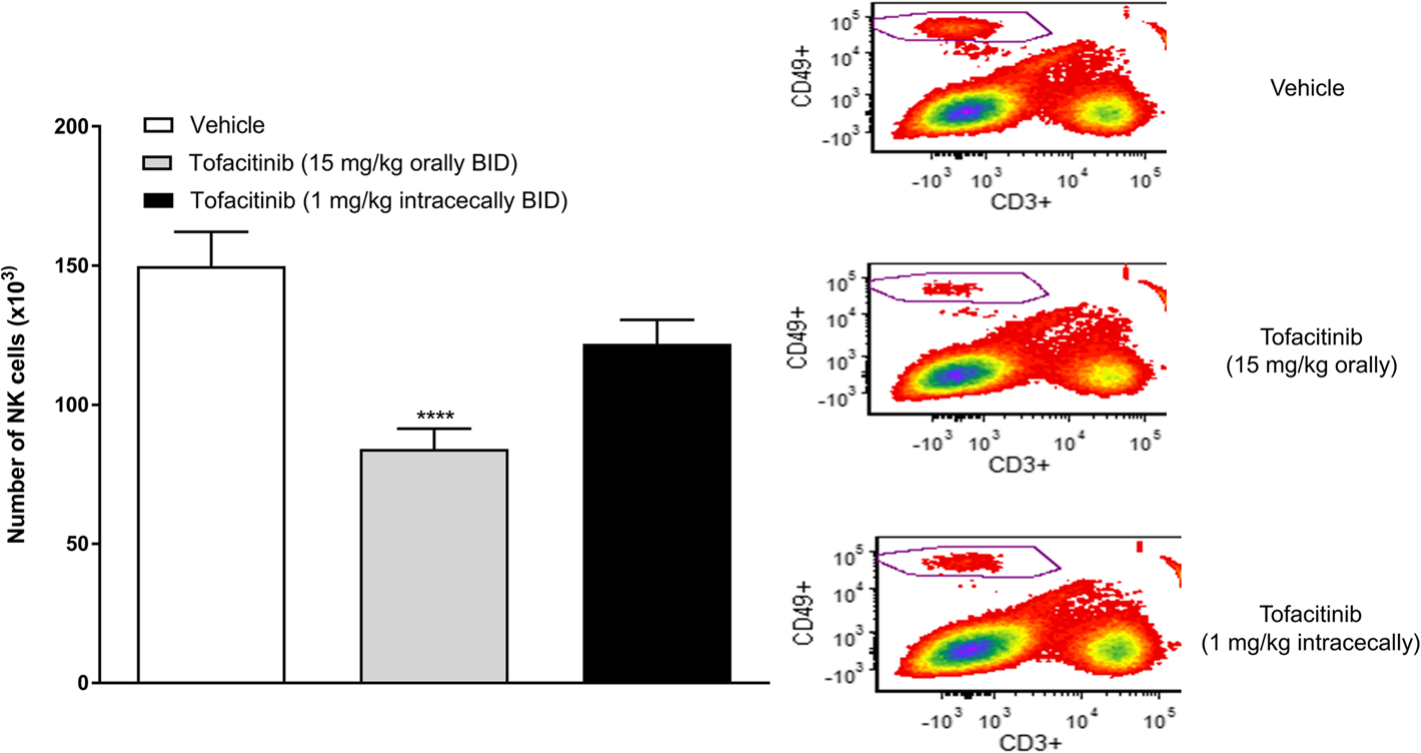
The effect of tofacitinib, dosed orally (15 mg/kg BID, with accompanying intracecal vehicle dosing) or intracecally (1 mg/kg BID, with accompanying oral vehicle dosing) on the number of NK cells in the spleen. Each control mouse received both oral and intracecal vehicles. The mean values (± SEM) for each treatment group are shown (n = 9 or 10). **** p < 0.0001 vs. Vehicle (One-way ANOVA, Fisher’s post-hoc test). Representative flow cytometry plots of splenic lymphocytes (NK cells circled) are also shown

Tofacitinib, dosed orally (15 mg/kg BID), but not intracecally (1 mg/kg BID), produced a statistically significant reduction in splenic NK cell counts (by 44%), compared to the vehicle-treated mice (Fig. 8). The mean (±SEM) plasma and spleen C_max_ values (*n* = 3) for tofacitinib were 979 ± 156 ng/mL and 1342 ± 200 ng/g, respectively, following oral dosing, and 23 ± 5 ng/mL and 33 ± 11 ng/g, respectively, following intracecal dosing.

## Conclusions

The demonstrated efficacy of the JAK inhibitor, tofacitinib, in Phase 2 and Phase 3 clinical studies in ulcerative colitis patients supports the potential of this class of agents in providing much needed benefit to IBD patients [17, 18]. However, due to its high small intestinal absorption [29, 30], systemic JAK inhibition by tofacitinib can be anticipated, and the resulting immunosuppression may lead to an increased risk of infection and malignancy as observed in rheumatoid arthritis patients [19, 20]. The data from this preclinical study suggest that restricting the activity of a JAK inhibitor to the intestine could confer clinical efficacy in IBD patients, in the absence of systemic adverse effects. However, although the murine oxazolone-induced colitis model shares many features of human ulcerative colitis [21, 22], the use of a hapten (i.e., oxazolone) to induce an acute colitis is clearly different from a disease of multifactorial etiology, and a chronic waxing and waning presentation in patients.

Orally dosed tofacitinib (10 and 15 mg/kg TID) produced a reduction in the DAI score, and its associated subscores (i.e., changes in body weight, stool consistency and blood content). Interestingly, at a higher dose (30 mg/kg TID), tofacitinib lacked activity. While the reason for the apparent bell-shaped dose-response curve to tofacitinib is unclear, this observation is not unique to a JAK inhibitor as it has also been noted with a bifunctional IL-4/IL-13 antagonist [31], and with the steroid, prednisolone (Theravance Biopharma US, Inc., unpublished observation). The efficacy of intracecally dosed tofacitinib (1 mg/kg BID) was comparable to that of the 15 mg/kg BID oral dose of tofacitinib, while the drug exposure profiles were strikingly different. Intracecal dosing of tofacitinib resulted in colon exposures that were similar to a 15-fold higher oral dose, but markedly lower plasma exposures (77-fold), resulting in a higher colon:plasma exposure ratio. This finding is presumably related to the absence of absorption in the small intestine following direct administration of the drug to the cecum. In conclusion, the pharmacokinetic profiles indicate that tofacitinib-induced inhibition of oxazolone-evoked colitis correlated with colonic, rather than plasma drug exposure. These data imply that local JAK inhibition in the colon, in the absence of appreciable systemic exposure, is sufficient to achieve efficacy in the oxazolone model of colitis. Moreover, the efficacious oral, but not intracecal, dose of tofacitinib in the oxazolone model attenuated splenic NK cell counts, a sensitive measure of systemic JAK inhibition [23, 24]. These data support the concept of an improved therapeutic index for a JAK inhibitor upon localized intestinal exposure.

Pharmacodynamic data in this study provided further support for direct JAK inhibition by tofacitinib in the mouse colon. Tofacitinib, dosed orally or intracecally, was associated with an inhibition of oxazolone- or IFNγ-induced elevation of colonic pSTAT1 levels, consistent with localized JAK inhibition. Elevated intestinal levels of IFNγ have been demonstrated in biopsies from IBD patients [32]. Measurement of colonic tofacitinib concentrations in the pharmacodynamic studies suggested that there would have been significant inhibition of IFNγ- and oxazolone-induced tissue pSTAT1 levels at maximally efficacious doses (i.e., 15 mg/kg orally, and 1 mg/kg intracecally BID) in the oxazolone colitis model. Notably, oral doses of tofacitinib required for inhibition of oxazolone-induced pSTAT1 elevation were lower than those required for efficacy in the oxazolone colitis model. The reduced oral potency of tofacitinib in the disease model may reflect a requirement to inhibit multiple, and more distal JAK/STAT-mediated events in an inflammatory cascade, rather than preventing a single proximal JAK/STAT pathway by oxazolone-induced cytokine release.

In order to isolate the colon from any potential systemic influences of cytokine stimuli or tofacitinib recirculation, the mouse colon was subjected to luminal application of the JAK inhibitor in an organ bath setting. In this preparation, tofacitinib inhibited the IFNγ-induced pSTAT1 response (mean IC_50_ of 3.1 μM). This observation indicates that in an in vitro setting, when its concentration is maintained luminally, tofacitinib is able to inhibit JAK activity in the colon directly.

In conclusion, the data from this study demonstrate inhibition of JAK locally in the intestine, and suggest that this approach may have clinical potential for patients with IBD. As this conclusion is based on the use of a single rodent disease model, which is unlikely to duplicate precisely colitis in patients, further preclinical and clinical investigation of this concept is warranted. The ability to separate the desirable intestinal, from undesirable systemic, activities of a JAK inhibitor is appealing, although it is unclear on the basis of its high inherent absorption [29, 30], whether a formulation of tofacitinib, or any other currently available JAK inhibitor, could be developed to provide a clinically meaningful degree of intestinal selectivity. Ideally, a compound has to reach, and enter, the epithelial cells and/or immune cells in the lamina propria from the intestinal lumen, to inhibit the JAK enzyme, and yet have limited absorption into the systemic circulation. The development of a novel JAK inhibitor chemically designed to be poorly absorbed and intestinally restricted to achieve targeted JAK engagement in the intestinal mucosa may be a more propitious approach.
